# Development and validation of a prognostic index for efficacy evaluation and prognosis of first-line chemotherapy in stage III–IV lung squamous cell carcinoma

**DOI:** 10.1007/s00330-018-5912-2

**Published:** 2019-01-14

**Authors:** Jiangdian Song, Jie Tian, Lina Zhang, Xiujuan Qu, Wei Qian, Bin Zheng, Lina Zhang, Jia Zhao, Meng Niu, Mu Zhou, Lei Cui, Yunpeng Liu, Mingfang Zhao

**Affiliations:** 1 School of Medical Informatics, China Medical University, Shenyang, Liaoning China; 2 Department of Medical Oncology, The First Hospital of China Medical University, Shenyang, 110001 Liaoning China; 3 CAS Key Laboratory of Molecular Imaging, Institute of Automation, Chinese Academy of Sciences, Beijing, China; 4 College of Engineering, University of Texas, El Paso, TX USA; 5 Sino-Dutch Biomedical Engineering School, Northeastern University, Shenyang, Liaoning China; 6 Medicine and Biological Information Engineering, University of Oklahoma, Norman, OK USA; 7 Department of Radiology, The First Hospital of China Medical University, Shenyang, Liaoning China; 8 Department interventional therapy, The First Hospital of China Medical University, Shenyang, Liaoning China; 9 Artificial Intelligence in Medicine and Imaging (AIMI) Center, Department of Radiology, Stanford University, San Francisco, CA USA

**Keywords:** Biomarkers, Tumor, Carcinoma, Squamous cell, Prognosis

## Abstract

**Objectives:**

To establish a pre-therapy prognostic index model (PIM) of the first-line chemotherapy aiming to achieve accurate prediction of time to progression (TTP) and overall survival among the patients diagnosed with locally advanced (stage III) or distant metastasis (stage IV) lung squamous cell carcinoma (LSCC).

**Methods:**

Ninety-six LSCC patients treated with first-line chemotherapy were retrospectively enrolled to build the model. Fourteen epidermal growth factor receptor (*EGFR*)-mutant LSCC patients treated with first-line EGFR-tyrosine kinase inhibitor (TKI) therapy were enrolled for validation dataset. From CT images, 56,000 phenotype features were initially computed. PIM was constructed by integrating a CT phenotype signature selected by the least absolute shrinkage and selection operator and the significant blood-based biomarkers selected by multivariate Cox regression. PIM was then compared with other four prognostic models constructed by the CT phenotype signature, clinical factors, post-therapy tumor response, and Glasgow Prognostic Score.

**Results:**

The signature includes eight optimal features extracted from co-occurrence, run length, and Gabor features. By using PIM, chemotherapy efficacy of patients categorized in the low-risk, intermediate-risk, and high-risk progression subgroups (median TTP = 7.2 months, 3.4 months, and 1.8 months, respectively) was significantly different (*p* < 0.0001, log-rank test). Chemotherapy efficacy of the low-risk progression subgroup was comparable with EGFR-TKI therapy (*p* = 0.835, log-rank test). Prognostic prediction of chemotherapy efficacy by PIM was significantly higher than other models (*p* < 0.05, *z* test).

**Conclusion:**

The study demonstrated that the PIM yielded significantly higher performance to identify individual stage III–IV LSCC patients who can potentially benefit most from first-line chemotherapy, and predict the risk of failure from chemotherapy for individual patients.

**Key Points:**

*• TTP and OS of first-line chemotherapy in individual stage III–IV LSCC patients could be predicted by pre-therapy blood-based biomarkers and image-based signatures.*

*• Risk status of pre-therapy indicators affected the efficacy of first-line chemotherapy in stage III–IV LSCC patients.*

*• Those stage III–IV LSCC patients who were able to achieve similar efficacy to EGFR-TKI therapy through chemotherapy were identified.*

**Electronic supplementary material:**

The online version of this article (10.1007/s00330-018-5912-2) contains supplementary material, which is available to authorized users.

## Introduction

The number of newly increased lung cancer cases per year is about 1.8 million worldwide [1]. According to official statistics released by the World Health Organization, among those, approximately 20~30% are lung squamous cell carcinoma (LSCC) [2]. Although the proportion of LSCC is lower than lung adenocarcinoma, due to the lack of drugs targeting oncogenic drivers, and the contraindication of approved drugs (bevacizumab and pemetrexed), median overall survival (OS) of the locally advanced (stage III) and distant metastasis (stage IV) LSCC patients is less than 1 year [3, 4]. The unsatisfactory prognosis has now becoming a major challenge in clinical treatment of stage III–IV LSCC patients. Chemotherapy is still the preferred treatment of stage III–IV LSCC patients at present according to the clinical practice guideline of American National Comprehensive Cancer Network (NCCN) [5].

Gemcitabine-cisplatin (or carboplatin), paclitaxel-cisplatin (or carboplatin), and docetaxel-cisplatin (or carboplatin) are the proven effective chemotherapy regimens to LSCC patients in current clinical practice [3, 5, 6]. It is noteworthy that although there are various chemotherapy regimens, some patients are still unable to benefit from chemotherapy, and tumor progression could be soon detected by imaging-based examination [7, 8]. On the other hand, although stage III–IV LSCC patients were pathologically consistent, distinct tumor heterogeneity of these patients with the same pathological subtype potentially herald disparate long-term clinical outcome [9, 10]. However, how to assess and quantify the pre-therapy tumor heterogeneity in these patients in a less traumatic or non-invasive way, so as to predict the risk of disease progression, and evaluate the tumor response to chemotherapy in the individual patient and estimate the long-term survival difference in different tumor heterogeneity groups, is still unexplored.

Recently, as a new emerging technology in medical imaging informatics, quantitative analysis method proposes to extract high-throughput imaging features from the medical images of tumors and subsequently construct a predictive model for the clinical purpose of auxiliary diagnosis or prognosis prediction [11–13]. With advanced image feature analysis, heterogeneity of tumor on medical images is converted into mineable high-dimensional data [14, 15]. Studies have shown that intra-tumor heterogeneity had higher impact on individualized treatment outcome. The patients with homologous tumor manifestations trend to express similar clinical outcomes [9, 16, 17], while the patients diagnosed with distinct tumor heterogeneity, potentially driven by different biological processes, ultimately result in varied clinical outcome [18, 19]. The quantitative high-dimensional features provide rich information on the tumor phenotype and can potentially be used to evaluate the prognosis of chemotherapy in LSCC patients. Previous studies of CT data have achieved promising performance in predicting the clinical outcome of LSCC patients [11, 20], indicating the potential of applying quantitative heterogeneity analysis to the efficacy evaluation and prognosis of chemotherapy in stage III–IV LSCC patients.

Previous studies have confirmed that the blood-based biomarkers were the key factors for cancer prognosis [21–25]. In this study, we hypothesized that the integration of blood-based biomarkers and pre-therapy CT image-based heterogeneity might provide more potential valuable prognostic information to stage III–IV LSCC patients treated with first-line chemotherapy. In order to validate this hypothesis, we analyzed the potential relationship between high-throughput pre-therapy CT phenotype features and time to progression (TTP) in stage III–IV LSCC patients. Specifically, we integrated the CT phenotype features and blood-based biomarker into a prognostic index model (PIM) to predict the risk of progression for individual stage III–IV LSCC patient. The effectiveness of PIM was validated by a cohort of stage III–IV epidermal growth factor receptor (*EGFR*)-mutant LSCC patients who only received first-line EGFR-tyrosine kinase inhibitor (TKI) therapy. To show the potential advantages of PIM, its performance was compared with other four prognostic prediction models, and the feasibility of applying the PIM to OS prognosis in stage III–IV LSCC patients was also explored.

## Materials and methods

### Patients

Eligibility criteria for this study were the following: diagnosed with stage III–IV LSCC from January 2013 to December 2017, age older than 20 years, received first-line chemotherapy or first-line EGFR-TKI therapy according to the criteria established by the clinical guidelines, with pre-therapy blood test and biochemical results, pre-therapy contrast-enhanced CT images were acquired in 2 weeks before chemotherapy, tumor response was evaluated by chest CT examination according to the RECIST1.1 standard at the end of every 2 cycles of admission of chemotherapy, with ECOG performance status (PS) score of 0 to 2, with normal organ function, and with no surgery resection history. Finally, 96 stage III–IV LSCC patients received first-line chemotherapy, and 14 stage III–IV LSCC patients with confirmed EGFR-positive mutation who received first-line EGFR-TKI were eligible in this study. Detailed information of patients and treatment regimen is presented in Tables 1 and 2.

**Table 1:** Demographic information of the enrolled patients in this study

Variables	Chemotherapy patients	EGFR-TKI patients
Age		
< 65	86	8
≥ 65	10	6
Gender		
Male	82	3
Female	14	11
Smoking		
Yes	78	2
No	18	12
Family of history		
Yes	11	1
No	85	13
Clinical stage		
IIIA~IIIB	55	3
IV	41	11
ECOG PS score		
< 2	40	8
= 2	56	6

*ECOG PS* Eastern Cooperative Oncology Group performance status

**Table 2:** Treatment regimens and corresponding time to progression (TTP) of the enrolled patients in this study

Regimen	Dosage	Number	Median TTP (months)
GP	Gemcitabine (1.0 g/m^2^) plus cisplatin (75 mg/m^2^)	46	3.7
TC	Docetaxel (75 mg/m^2^) plus carboplatin (5 × (CCr + 25))	12	1.9
TP	Paclitaxel (135 mg/m^2^) plus cisplatin (75 mg/m^2^)	10	3.2
DP	Docetaxel (75 mg/m^2^) plus cisplatin (75 mg/m^2^)	11	3.2
Other	–	17	4.7
Gefitinib	250 mg/qd	14	5.2

*GP* gemcitabine-cisplatin, *TP* paclitaxel-cisplatin, *TC* docetaxel-carboplatin, *DP* docetaxel-cisplatin, *CCr* creatinine clearance rate

Patients underwent contrast-enhanced chest CT using a Siemens SOMATOM Definition Flash 64-row dual-source CT machine. Patient took a supine position and raised his arms, and lung was scanned at the end of inhalation. Parameters were as follows: tube voltage of 100 kV or 140 kV, tube current of Care Dose 4D, scanning layer thickness of 2 mm, reconstructed layer thickness of 2 mm, reconstructed layer spacing of 2 mm, matrix of 512 × 512, and FOV of 350 mm × 350 mm. The enhanced scan was performed by a double-barrel high-pressure syringe to inject 70 ml to 90 ml of the non-ionic contrast agent iopromide intravenously into the cubital vein. The injection speed is 2.5 ml/s to 3.0 ml/s, and arterial phase images are obtained after 30 s to 40 s of injection.

TTP was the primary endpoint, and OS was the secondary endpoint in this study. Patients with chemotherapy were reviewed every 3 weeks, and the follow-up interval was 2–6 weeks in patients with EGFR-TKI therapy. TTP was considered the time from the initiation of therapy to the date of confirmed disease progression or death. OS was considered the time from the initiation of therapy to the date of death. Median follow-up of chemotherapy was 11.1 months in this study. Patients were censored if they were alive at the last follow-up or were lost to follow-up. This study was approved by the institutional review board and ethics committee of the First Affiliated Hospital of China Medical University and carried out in accordance with the Declaration of Helsinki.

### Image-based prognostic signature building

CT scans, clinical demographics, and blood-based information for all patients were collected together for unified record and standardized storage in this study. The region of interest (ROI) of primary tumor of the chemotherapy patients on CT images was manually segmented by two radiologists with more than 10 years of experience in thoracic radiology. All radiologists have received thoracic training, and any disagreements were resolved in a consensus meeting with other radiologists and oncologists. For each patient, 356 three-dimensional phenotypic features and 236 two-dimensional phenotypic features were automatically extracted on the tumor ROI by C++ program. Based on the feature matrix consisted of a total of 56,000 CT phenotype features which were extracted from the 96 chemotherapy patients, the features were evaluated by the following two steps: first, the prognostic value of all the features for TTP was evaluated by univariate Cox analysis. Then, the features identified as significant (*p* < 0.05) in univariate Cox analysis were subsequently fed into the least absolute shrinkage and selection operator (LASSO) Cox regression to build an image-based prognostic signature. Patients with different signature scores would be classified into different groups according to the optimal cut-off value by X-tile, which was a widely recognized tool for calculating optimal cut-off values (Yale University School of Medicine) [26]. For details, please see supplementary part 1.

### PIM construction and validation

PIM was constructed by the significant clinical prognosticators and the image-based phenotypic signature, as described by the following steps: first, in order to select the significant prognostic clinical variables, 24 clinical variables were evaluated by univariate Cox regression analysis, including seven demographics features (namely the sex, age, ECOG, number of smoke, smoke status, history of disease, family history), three clinical features (T, N, M stage), and 14 blood-based variables, as described in Table 3 and supplementary Table S1. Cut-off values of the variables of demographic, clinical, and blood indicators were determined according to previous studies [27] or current clinical practice in order to transform them into normal status or risk status for univariate Cox regression analysis and PIM construction.

**Table 3:** Univariate Cox regression of the 24 clinical and blood-based biomarkers according to the primary endpoint of time to progression

Factors	*β*	Wald	HR	95% CI	*p* value
Gender	0.20	0.35	1.22	0.63–2.34	0.56
Age	− 0.02	0.004	0.98	0.58–1.73	0.95
EOCG	0.11	0.05	1.11	0.45–2.73	0.82
Number of smoke	− 0.25	1.01	0.78	0.47–1.27	0.31
Smoke status	0.12	0.13	1.13	0.60–2.11	0.72
History of lung cancer	0.16	0.35	1.17	0.70–1.95	0.55
Family history	0.92	3.57	2.50	0.97–6.45	0.06
WBC	0.35	1.40	1.42	0.79–2.55	0.24
NE	− 0.02	0.003	0.98	0.56–1.72	0.95
LY	− 0.26	0.66	0.77	0.42–1.44	0.42
MONO	− 0.19	0.57	0.83	0.51–1.35	0.45
EO	0.21	0.29	1.23	0.58–2.59	0.59
HB	0.0001	0.001	1.00	0.56–1.79	0.99
PLT	0.32	0.79	1.38	0.68–2.82	0.37
ALT	0.73	3.98	2.08	1.01–4.26	0.03*
TBIL	− 0.46	0.59	0.63	0.20–2.04	0.44
ALB	− 0.05	0.02	0.95	0.49–1.85	0.89
AST	1.17	13.14	3.22	1.71–6.04	< 0.0001*
FG	0.11	0.120	1.11	0.60–2.06	0.73
TP	− 0.22	0.67	0.81	0.48–1.35	0.41
CEA	0.76	6.21	2.13	1.18–3.85	0.01*
T stage	− 0.01	0.002	0.99	0.56–1.74	0.96
N stage	0.26	0.53	1.30	0.64–2.65	0.47
M stage	0.02	0.007	1.02	0.63–1.67	0.93

The median of the number of smoke (9600 cigarettes) was used as the cut-off value. TNM stage was divided into three variables for analysis

*WBC* white blood cell, *NE* neutrophil, *LY* lymphocyte, *MONO* monocytes, *EO* eosinophils, *HB* hemoglobin, *PLT* platelet, *ALT* alanine aminotransferase, *TBIL* total bilirubin, *ALB* albumin, *AST* aspartate aminotransferase, *FG* fibrinogen, *TP* total protein, *CEA* carcinoembryonic antigen

*The factor is significantly associated with time to progression

Next, the significant variables in univariate Cox regression analysis and the image-based prognostic signature in the previous section were fed into multivariable Cox regression analysis. The independent significant variables in multivariable Cox regression were then identified and used as PIM indices for model construction. For each patient, if all the PIM indices were at normal status, his/her PIM score was assigned a value of 0; if only one PIM index was at risk status, the patient’s PIM score was assigned a value of 1; if two PIM indices were at risk status, the patient’s PIM score was assigned a value of 2, and so on. Finally, the PIM we built in this study stratified all the chemotherapy patients into three progression risk subgroups: low-risk (PIM score = 0), intermediate-risk (PIM score = 1), and high-risk (PIM score ≥ 2).

After the PIM was trained and built using the data acquired from 96 patients, data acquired from other 14 *EGFR*-mutant stage III–IV LSCC patients who received first-line EGFR-TKI therapy were included to further validate the accuracy of chemotherapy efficacy prediction by the PIM.

### Accuracy comparison of the TTP prediction

In this section, the significant clinical and blood-based variables in univariate Cox regression model were used to perform multivariable Cox regression analysis and build a clinical model to compare with the proposed PIM model. In accordance with the same stratification standard, the chemotherapy patients were stratified into three risk subgroups by the clinical model (risk factor = 0, or 1, or ≥ 2). Besides, patients with both records of C-reactive protein (cut-off value 10 mg/L) and albumin (cut-off value 35 g/L) were used to build the Glasgow Prognostic Score (GPS) [28]. Another model based on the proposed pre-therapy signature was built to divide the chemotherapy patients into three risk subgroups by X-tile. In addition, a model based on the tumor response measured after chemotherapy (complete response (CR), partial response (PR), stable disease (SD), and progressive disease (PD)) was built for chemotherapy efficacy prediction. All the four methods mentioned above were employed for progression risk prediction, and the accuracy was compared with the PIM in this study.

### OS prognostication by the PIM

To further explore the survival prognostic utility of the PIM, we applied it to OS prognostication. The model-built details in this experiment were consistent with those described in the previous two sections.

Additionally, an ad hoc analysis was performed to test the prognostic efficacy of clinical and blood-based variables for the endpoint of OS by Cox regression analysis.

### Statistical data analysis

Statistical analysis was conducted using R software (version 3.2.3). Parameters of the packages in R used in this study are described in supplementary part 3. The Kruskal-Wallis test was used to evaluate the difference of demographics variables in the three risk subgroups. The reported statistical significance levels were all two-sided, and *p* values < 0.05 were considered to indicate significance.

For different subgroups stratified by the models, hazard ratio (HR) was used to compare the difference of TTP among subgroups. The Kaplan-Meier survival curves (log-rank test) were used to calculate the survival curve rate and evaluate the statistical significance of differences.

Harrell’s concordance index (C-index) [29] was used for quantifying the prognosis accuracy of the models. Nomogram of the models was also established to evaluate their prognosis performance [30]. Decision curve analysis was performed for comparing the net benefits at different threshold probabilities given by the models [31]. The net reclassification improvement (NRI) and the integrated discrimination improvement (IDI) were also quantified for evaluating the prognostic benefit improvement of the PIM.

## Results

**Fig. 1:**
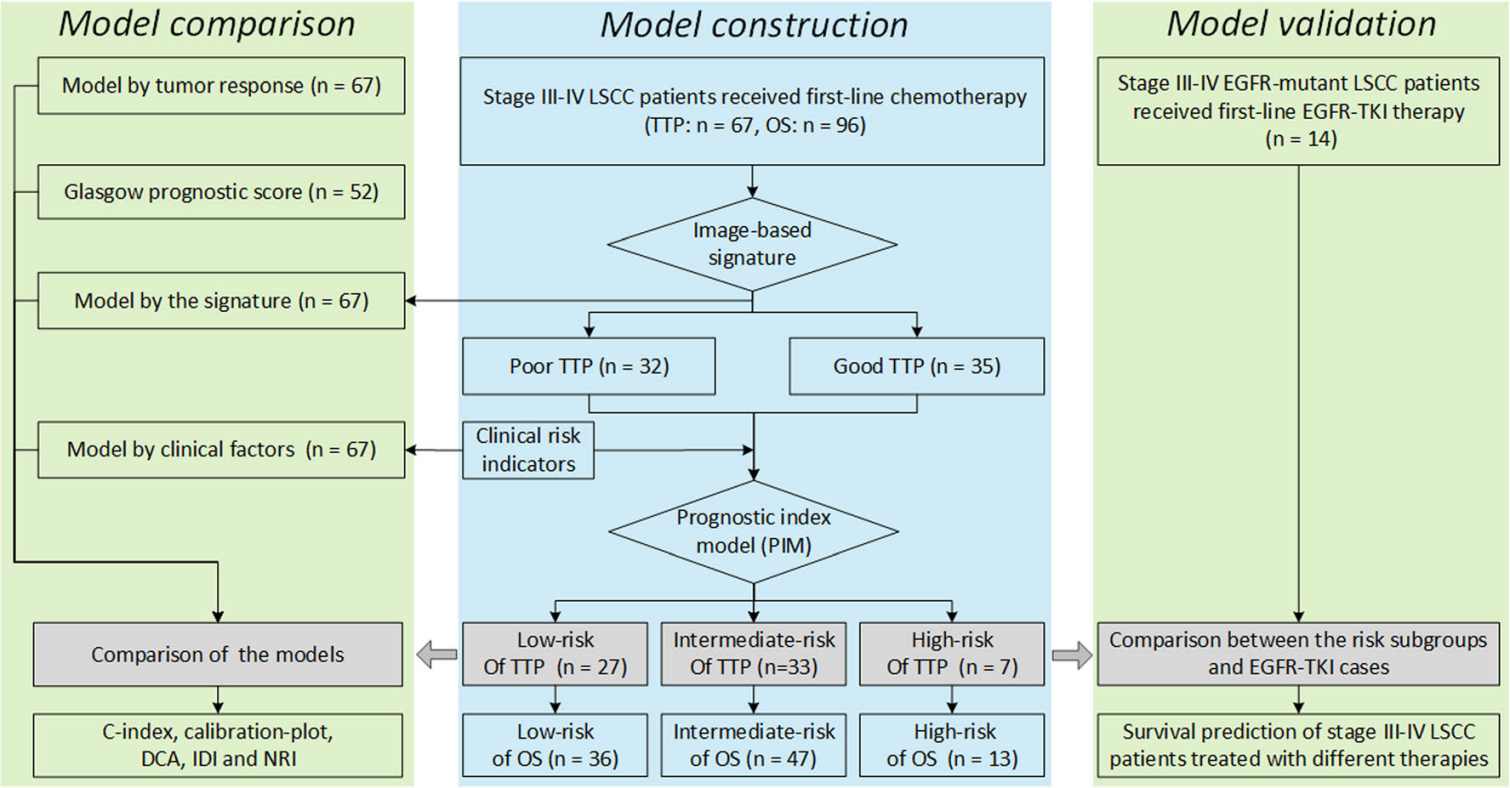
Flowchart of this study. The first step was model construction, and based on the constructed model, model validation and comparison were performed. LSCC, lung squamous cell carcinoma; TTP, time to progression; OS, overall survival. NRI net reclassification improvement, IDI integrated discrimination improvement

**Fig. 2:**
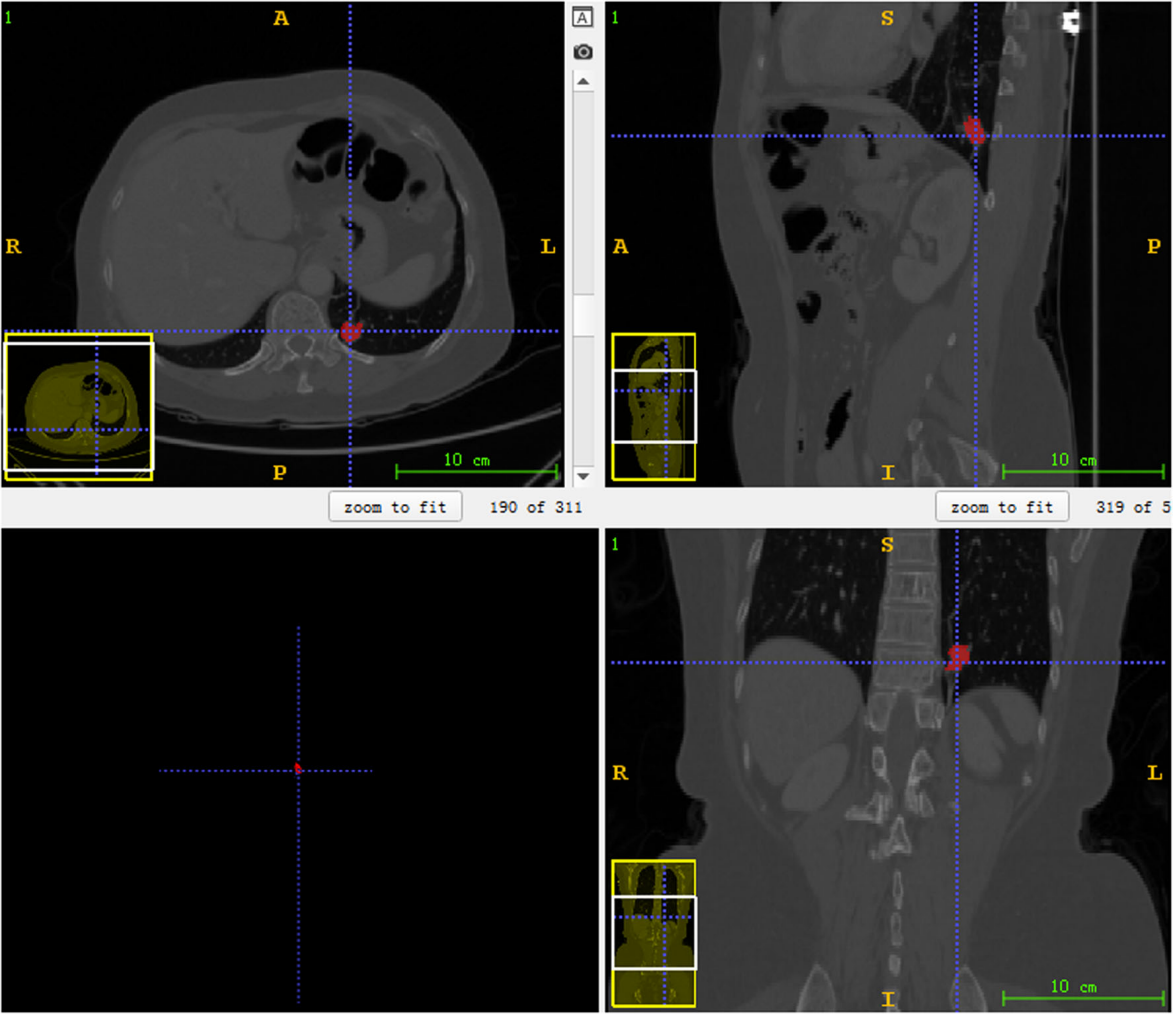
The diagram of manual segmentation by using ITK-SNAP. The subgraph in the upper left corner indicates that the manually segmented region of interest (ROI) by the radiologist from cross section. The subgraphs in the upper right and lower right corners represent the manual segmentation result of the tumor which is displayed from the sagittal and coronal planes, respectively. The tumor is then reconstructed in a view of three dimensions, which is represented in the subgraph in the lower left corner. Each of the subgraphs could be scaled to ensure accurate segmentation

The flow chart of this study is shown in Fig. 1. Among the 110 stage III–IV LSCC patients enrolled in this retrospective study, seven patients with unqualified segmentation results and four patients with segmentation data unable to recognize were required to re-segment after the blind review, until qualified. Figure 2 describes the manual segmentation by using ITK-SNAP [32].

The median TTP and OS of the chemotherapy patients were 3.60 and 11.50 months, respectively. The median TTP of the EGFR-TKI patients was 5.20 months. A significant difference of demographics variables was not found in the subgroups classified by the signature and the PIM (*p* > 0.05). All the chemotherapy patients had a detailed follow-up of OS (in 19 censored cases, three patients were lost during the OS follow-up and 16 patients were still alive at the last follow-up in this study), and 67 patients had a confirmed document of disease progression after chemotherapy (no censored data). Patients in our study were classified into CR (*n* = 0), PR (*n* = 27), SD (*n* = 56), and PD (*n* = 13) according to the RECIST1.1.

**Fig. 3:**
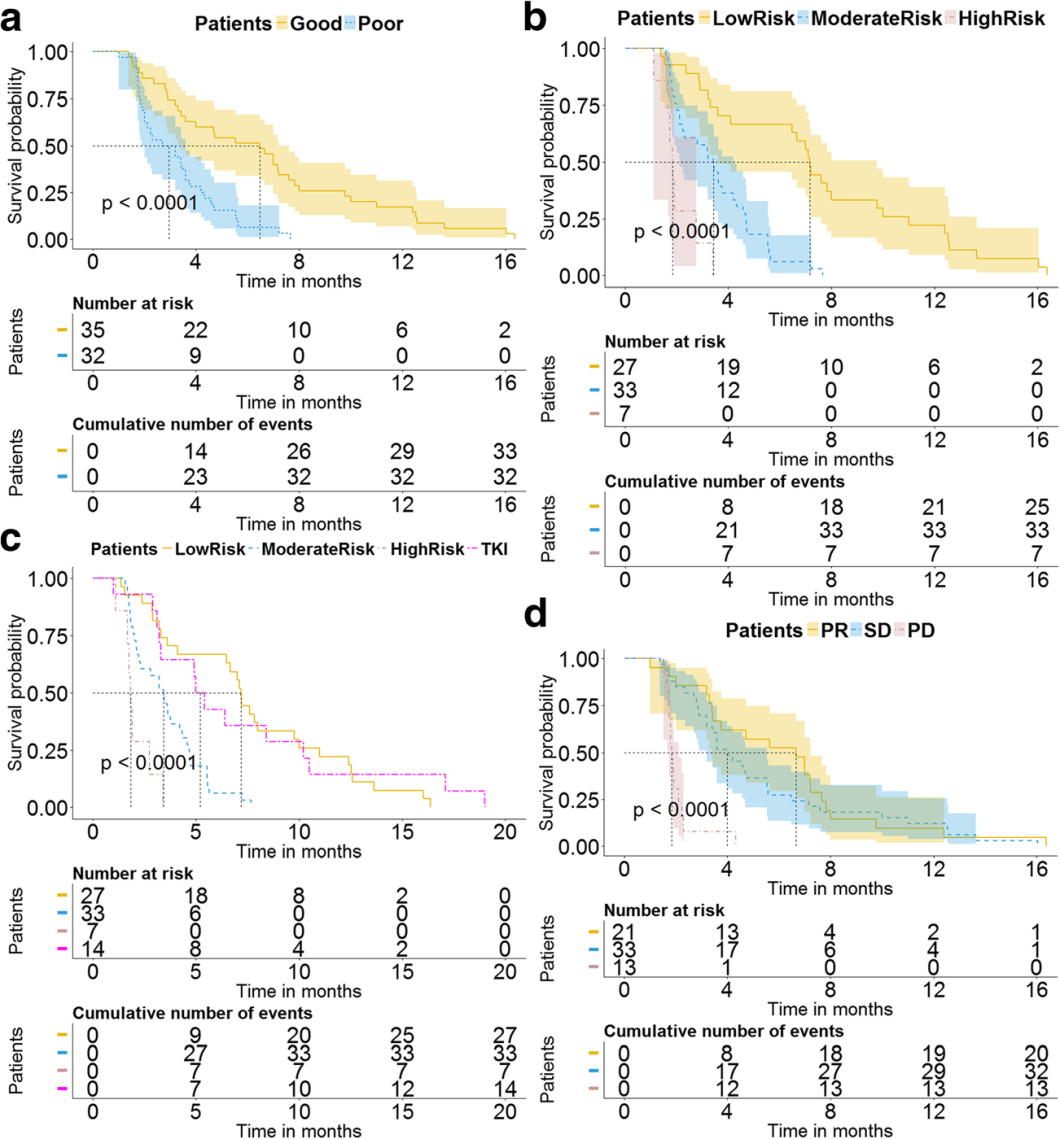
Results of progression risk prediction. **a** The Kaplan-Meier curves of groups classified by the signature, and all patients were stratified into good time to progression (TTP) group and poor TTP group according to the signature. **b**, **c** The progression risk prediction of the prognostic index model (PIM). **b** The result of low-risk (yellow line), intermediate-risk (blue line), and high-risk (pink line) progression subgroups by the PIM. **c** The comparison between the stage III–IV *EGFR*-mutant LSCC patients treated with first-line EGFR-TKI therapy (red line) and the different risk subgroups of chemotherapy patients stratified by the PIM. **d** The Kaplan-Meier curves of the patients with partial response (PR), stable disease (SD), and progressive disease (PD)

Eight phenotypic features, which include co-occurrence, run length, and Gabor features, and their corresponding weights are displayed in Eq. 1 (for a detailed description, please see supplementary). Cut-off value of the signature was − 1.117. In patients with a score lower than the cut-off value, his/her TTP benefit tended to be better (median TTP = 6.7 months), and these patients had higher scores indicating faster progression (median TTP = 3.2 months) in this study (HR = 2.45, 95% CI = 1.44–4.23, *p* < 0.0001), as presented in Fig. 3a.

1 $$ {\displaystyle \begin{array}{l}\mathrm{Signature}=5.65412e-11\times \mathrm{Value}\ \mathrm{of}\ \mathrm{cluster}\ \mathrm{shade}\ \mathrm{of}\ \mathrm{co}-\mathrm{occurrence}\ \left[0,1\right]\\ {}-2.726245e-04\times \mathrm{Value}\ \mathrm{of}\ \mathrm{variance}\ \mathrm{of}\ \mathrm{first}-\mathrm{order}\ \mathrm{feature}\left[1\right]\\ {}-1.980131e+01\times \mathrm{Value}\ \mathrm{of}\ \mathrm{low}\ \mathrm{gray}-\mathrm{level}\ \mathrm{run}\ \mathrm{emphasis}\ \mathrm{of}\ \mathrm{run}\ \mathrm{length}\ \left[3,4\right]\\ {}-3.909510e-03\times \mathrm{Value}\ \mathrm{of}\ \mathrm{GMTR}\ \mathrm{variance}\ \mathrm{of}\ \mathrm{GW}\ \mathrm{feature}\ \left[8\right]\\ {}-1.039332e-17\times \mathrm{Value}\ \mathrm{of}\ \mathrm{GMTR}\ \mathrm{variance}\ \mathrm{of}\ \mathrm{GW}\ \mathrm{feature}\ \left[12\right]\\ {}-1.021795e-05\times \mathrm{Value}\ \mathrm{of}\ \mathrm{GMTR}\ \mathrm{variance}\ \mathrm{of}\ \mathrm{GW}\ \mathrm{feature}\ \left[24\right]\\ {}+6.648282e-02\times \mathrm{Value}\ \mathrm{of}\ \mathrm{GPT}\ \mathrm{rentropy}\ \mathrm{of}\ \mathrm{GW}\ \mathrm{feature}\ \left[26\right]\\ {}-5.733282e-11\times \mathrm{Value}\ \mathrm{of}\ \mathrm{GMTR}\ \mathrm{variance}\ \mathrm{of}\ \mathrm{GW}\ \mathrm{feature}\ \left[28\right]\end{array}} $$

Univariate Cox regression analysis based on the chemotherapy patients indicated that the variables age, sex, ECOG, smoking, etc., were not prognostic significantly, except for aspartate aminotransferase (AST), alanine aminotransferase (ALT), and carcinoembryonic antigen (CEA) (*p* < 0.05), as presented in Table 3.

According to multivariable Cox regression analysis, the PIM was constructed by the following factors: the intra-tumor heterogeneity prognostic signature and AST, as shown in Table 4. According to the PIM, patients in the low-risk progression subgroup could be benefited most from first-line chemotherapy (median TTP = 7.2 months). However, when patients were classified into the intermediate-risk progression subgroup (median TTP = 3.4 months), the median TTP was reduced by nearly 120 days (*p* < 0.0001, HR = 2.57, 95% CI = 1.53–4.32). Furthermore, in patients classified into the high-risk progression subgroup (median TTP = 1.8 months), the median TTP was reduced by nearly 50 days compared with the intermediate-risk progression subgroup patients (*p* < 0.0001, compared with the other two subgroups), as shown in Fig. 3b.

**Table 4:** The results of the multivariable Cox regression analysis. Significant variables (*p* < 0.05) are used as prognostic indices to construct the prognostic index model

Variables	HR (95% CI)	*β*	*p* value
Signature	3.50 (1.88, 6.50)	1.25	< 0.0001
AST	3.66 (1.81, 7.39)	1.30	0.0003
ALT	1.69 (0.76, 3.78)	0.52	0.20
CEA	1.08 (0.55, 2.11)	0.07	0.82

*AST* aspartate aminotransferase, *ALT* alanine aminotransferase, *CEA* carcinoembryonic antigen

In the ad hoc analysis, AST and white blood cell (WBC) were the significant prognostic factors for the endpoint of OS by univariate Cox regression analysis, as presented in supplementary Table S2. Besides, the result of multivariate Cox regression analysis indicated that AST (HR = 1.79, 95% CI = 1.06–3.03, *p* = 0.02) was the only significant prognostic factor for OS in this experiment.

Significant TTP difference was not found between the stage III–IV *EGFR*-mutant LSCC patients treated with first-line EGFR-TKI therapy and the stage III–IV LSCC patients in the low-risk progression chemotherapy subgroup (*p* = 0.835, HR = 1.01, 95% CI = 0.60–1.72). But, the difference was significant when compared with that in the intermediate-risk or high-risk progression subgroups (*p* = 0.0025 and *p* = 0.0002, respectively), as described in Fig. 3c.

**Fig. 4:**
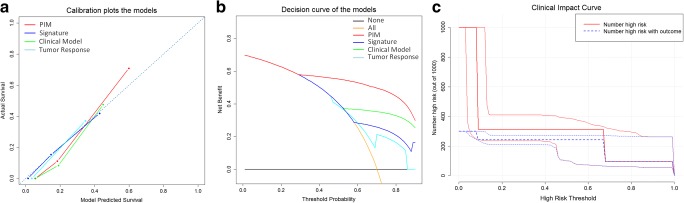
The comparison of the models. **a** The plots depict the calibration of the model in terms of the agreement between predicted and observed TTP (time.inc = 6 months). Performances of the models are shown on the plots relative to the 45° line, which represents perfect prediction. **b** Decision curve analysis of the PIM (red line), clinical factor-based model (green line), post-treatment tumor response-based model (cyan line), and the intra-tumor heterogeneity signature-based model (blue line). The orange line represents the assumption that all patients were treated. The black line represents the assumption that no patient was treated. The *x*-axis represents the risk of progression (Pt). The *y*-axis measures the net benefit. The net benefit was calculated by subtracting the proportion of all patients who are false positive from the proportion who are true positive, weighting by (Pt/(1 − Pt)). The decision curve showed that if the threshold probability of a patient or doctor is > 26%, using the PIM to predict progression risk adds more benefit than the treat-all-patients scheme or the treat-none scheme, or other prognostic models. **c** The clinical impact curve of the PIM; the red line (number of high risk) represents the patients with a high risk of progression predicted by the PIM at each threshold (with 95% CI), and the green line (number of high risk with outcome) represents the patients with actual progression at each threshold (with 95% CI)

According to the model based on the post-treatment tumor response, results indicated that a significant difference of TTP was found between the PD and disease control (PR and SD) patients (*p* < 0.0001, in both comparisons) but not found between the SD and PD patients (*p* = 0.40, HR = 1.25, 95% CI = 0.73–2.14), as presented in Fig. 3d. AST and CEA were indicated as the independent prognostic factors to construct the clinical factor-based model (*p* < 0.05). The difference of TTP in the three subgroups stratified by the clinical model was significant (see supplementary Fig. S1). However, the accuracy comparison of TTP prediction of the first-line chemotherapy indicated that the PIM outperformed that of all the other models (*p* < 0.05), as presented in Table 5. Decision curve analysis indicated that the prognostic performance of the PIM was significantly stronger than others (Fig. 4). According to the clinical impact curve of the PIM (Fig. 4c), when the probability of patient progression was greater than 10%, the prediction results of PIM were getting closer to actual situation.

**Table 5:** The comparison of the prognostic accuracy between the PIM and other four prognostic models (*z* test was used to calculate the *p* values by using the R package of “survIDINRI”)

Models	C-index (95% CI)	IDI (*p* value)	NRI (*p* value)
PIM	0.682 (0.649–0.715)	Ref	Ref
Clinical model	0.629 (0.600–0.658)	− 0.095 (0.013)	− 0.644 (0.013)
GPS	0.480 (0.433–0.527)	–	–
Signature	0.631 (0.600–0.662)	− 0.101 (< 0.001)	− 0.559 (< 0.001)
Tumor response	0.648 (0.613–0.683)	− 0.169 (0.020)	− 0.428 (0.027)

The IDI and NRI between PIM and GPS were blank as the patient population is different

*PIM* prognostic index model, *GPS* Glasgow Prognostic Score, *NRI* net reclassification improvement, *IDI* integrated discrimination improvement

**Fig. 5:**
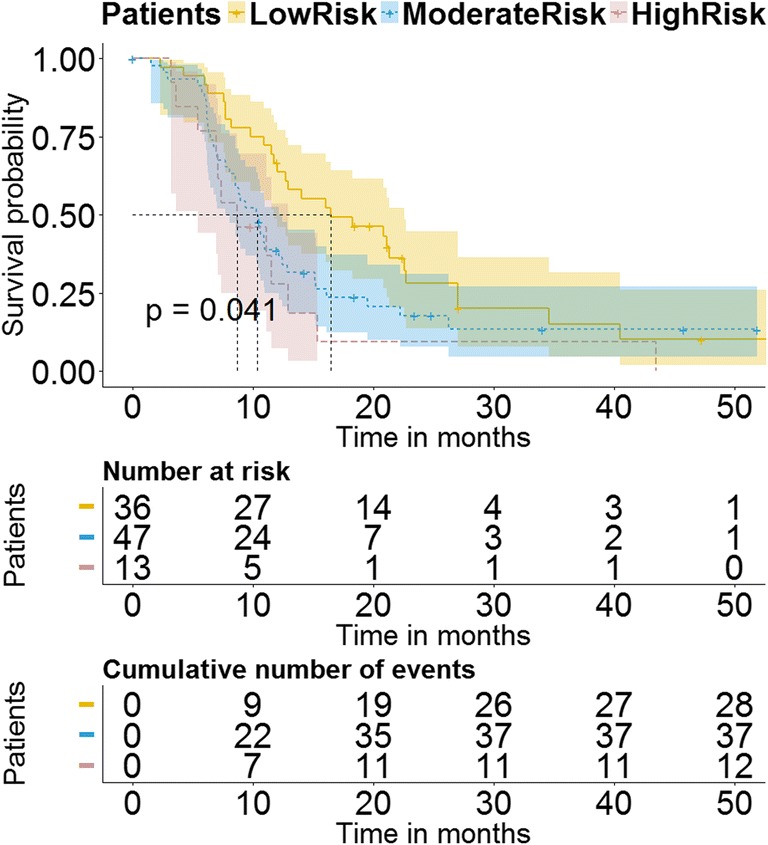
Prognostication of overall survival, a further exploration of the proposed PIM. By applying the PIM on overall survival, the Kaplan-Meier survival curves are the stratified high-risk (pink), intermediate-risk (blue), and low-risk (yellow) chemotherapy patient subgroups

OS prognostication by the PIM was performed on the 96 stage III–IV LSCC patients with first-line chemotherapy. Based on the same construction standard, OS of the low-risk progression subgroup stratified by PIM (median OS = 16.3 months) showed a substantial clear survival benefit compared with other patients (median OS = 9.6 months, HR = 0.58, 95% CI = 0.37–0.91, *p* = 0.02). The difference of OS was not statistically significant between the intermediate-risk and high-risk progression subgroups (*p* = 0.397), as shown in Fig. 5.

## Discussion

In this study, we proposed a pre-therapy non-invasive model for efficacy evaluation and long-term prognosis prediction of first-line chemotherapy in stage III–IV LSCC patients by the integration of blood test biomarkers and quantitative intra-tumor heterogeneity. Those individual stage III–IV LSCC patients who potentially benefited most from first-line chemotherapy, and the risk of failure from this therapy for individual stage III–IV LSCC patients were quantitatively evaluated.

Quantitative decoding of tumor heterogeneity on pre-therapy images could excavate potential tumor progression and prognostic information to aid clinical decision making [33, 34]. In this study, the progression risk of first-line chemotherapy in stage III–IV LSCC patients was accurately predicted according to the intra-tumor heterogeneity signature. This finding indicated that the critical chemotherapy-resistant information potentially hid in the high-dimensional tumor phenotype. An in-depth study of the phenotypic descriptors which express complex tumor heterogeneity might be more helpful to understand the mechanism of tumor progression [13].

To the best of our knowledge, this is the first study to integrate the pre-therapy tumor heterogeneity and blood-based biomarkers into an available tool for both TTP and OS prognosis for first-line chemotherapy in stage III–IV LSCC patients. According to the proposed PIM, our study identified the kind of stage III–IV LSCC patients who most likely to benefit from chemotherapy: outcomes of the low-risk progression subgroup patients (with low signature score and normal AST status) were significantly better than those of the other patients. The risk of progression in the low-risk progression subgroup patients was only 0.39 and 0.14 compared to that of the other two subgroup patients, and indeed 112% and 299% more TTP benefit than that of the two subgroup patients, respectively. Furthermore, OS of the low-risk progression subgroup patients was also significantly better than that of the other patients (*p* = 0.02). All of these findings consistently suggested that for stage III–IV LSCC patients, first-line chemotherapy was strongly recommended to those with a low risk of progression according to the PIM. In addition, by the comparison of the stage III–IV *EGFR*-mutant LSCC patients treated with first-line EGFR-TKI therapy, TTP of the low-risk progression subgroup was almost identical to that of the EGFR-TKI patients (*p* = 0.835). This finding was confirmed with previous studies that chemotherapy could achieve similar clinical efficacy as EGFR-TKI therapy for non-small cell lung cancer (NSCLC) patients [35–37]. As the observed incidence of EGFR mutations was only 2.7% in patients with LSCC, EGFR mutations were not applicable to routine testing of all LSCC tumor specimens [5]. Thus, early identification of the low-risk progression patient would be significantly vital for directing personalized therapeutic regimen administration, as well as achieving an optimized economic cost-to-benefit ratio for these patients.

According to our experiments, the clinical variables of sex, age, smoking status, and ECOG performance status were not significant prognosticators. Although these factors were widely concerned in the studies of NSCLC, we found that in previous reports, their prognostic performance was inconsistent in different NSCLC populations [35, 38–42]. Our finding was also suggested that current evidence was still inadequate to determine that particular types of NSCLC patient defined by age, sex, performance status, histology, or clinical stage could be benefited from chemotherapy [43]. On the other hand, studies to explore whether those factors could be used as independent prognostic factors of chemotherapy in advanced-stage LSCC patients are still rare. In addition, there are no preferred chemotherapy regimens for smoking LSCC patients for now [44]. More evidence is still needed to determine the prognostic value of the traditional clinical variables for stage III–IV LSCC patients.

GPS may not be suitable for the prognosis of chemotherapy in stage III–IV LSCC patients according to our results. The comparison of the PIM with the four prognostic methods demonstrated that the integration of pre-therapy intra-tumor heterogeneity signature and blood-based biomarkers could be more valuable in clinical practice. As a cancer-specific prognostic indicator, the prognostic value of AST has long been concerned [21–23]. As in clinical practice, unusual level of the AST often indicates abnormal liver function, and it was also the significant indicator of poor prognosis of first-line chemotherapy in stage III–IV LSCC patients according to this study. Actually, previous studies have demonstrated that the prognosis of advanced lung cancer patients with liver metastasis was the worst [45, 46]. Thus, we suspected that the abnormality in AST in patients with stage III–IV LSCC potentially indicated liver metastasis. The clinical factor-based prognostic model built in this study indicated that CEA was significantly related to prognosis of stage III–IV LSCC patients, but it was excluded in the PIM. We considered that the prognostic performance of CEA still should be further validated as also reported in studies [24, 25].

According to the PIM, the OS probability of the chemotherapy patients in the high-risk progression subgroup was poor than other patients (median OS = 8.7 months versus 11.7 months). As the high-risk progression subgroup patients benefited least from first-line chemotherapy (the worst TTP and OS), indicating that patients in this subgroup may not be suitable for first-line chemotherapy, and other alternative therapies should be considered.

Our study was limited by retrospective and lacks of prospective validation. The indistinguishable OS between the intermediate-risk and the high-risk progression subgroups may be due to the existence of censored data (see Fig. 5), and validation on larger dataset would be more convincing. Besides, EGFR-TKI therapy was the only comparative treatment in this study, and other treatments were not considered. Since the low incidence of the stage IIII–IV LSCC patients (only 20~30% in NSCLC) and the natural characteristics of EGFR mutation in this kind of population (only 2.7% in LSCC), the small number of patients in the validation dataset is another limitation. Multicenter validation trials in the future will narrow the bias caused by the patient population. The prognostic signature and the PIM will be further validated in future studies and analyzed the regularity of prognosis revealed by tumor heterogeneity, transforming the current studies of “exploration of relationship” in imaging heterogeneity to the studies of “recognition of regularity” for aiding clinical practice.

In conclusion, the proposed prognostic strategy can achieve accurate efficacy evaluation and prognosis prediction of first-line chemotherapy in individual stage III–IV LSCC patients, which holds promise to pre-therapy personalized therapeutic assistance for these patients.

## Electronic supplementary material

ESM 1 (XLSX 21 kb)

ESM 2 (DOCX 4825 kb)

## Abbreviations

ALT: Alanine aminotransferase
AST: Aspartate aminotransferase
CEA: Carcinoembryonic antigen
C-index: Harrell’s concordance index
CR: Complete response
GPS: Glasgow Prognostic Score
HR: Hazard ratio
IDI: Integrated discrimination improvement
LASSO: Least absolute shrinkage and selection operator
LSCC: Lung squamous cell carcinoma
NRI: Net reclassification improvement
OS: Overall survival
PD: Progressive disease
PIM: Prognostic index model
PR: Partial response
SD: Stable disease
TTP: Time to progression
WBC: White blood cell
