# Supplementary material for: Development and validation of a prognostic index for efficacy evaluation and prognosis of first-line chemotherapy in stage III–IV lung squamous cell carcinoma
Source: Eur Radiol. 2019 Jan 14;29(5):2388–98. doi: 10.1007/s00330-018-5912-2 (PMC6443600; doi:10.1007/s00330-018-5912-2)
Supplement: Supplementary file 2 — (DOCX 4825 kb) [file 330_2018_5912_MOESM2_ESM.docx]

**Supplementary material**

**Part 1: Pre-therapy CT images.**

The prognostic signature was calculated from the pre-therapy computer tomography (CT) images of chemotherapy patients in this study. All the CT images were acquired in two weeks before the start of chemotherapy.

CT scans, clinical demographics and blood-based information for all patients were collected together for unified record and standardized storage in this study. Pre-therapy CT images and clinical and blood test indices of the chemotherapy patients were interpreted qualitatively and quantitatively by two well-trained radiologist and oncologist, respectively. Standardized reporting forms were used to record clinic pathological and biochemical indices. Then, all CT images were performed tumor segmentation. Primary tumor of all chemotherapy patients was manually segmented by two radiologists with more than 10 years of experience in clinical radiology. All radiologists have received thoracic training, and any disagreements were resolved in a consensus meeting with other radiologists and oncologists. Manual segmentation was performed based on the ITK-Snap [1], which allowed point-by-point identification of tumor boundary. After all the manual segmentation were finished, the data was blind reviewed by our radiologists. Those unqualified or unrecognized segmentation data were required to re-segment, until qualified.

Feature extraction was then performed on the segmented tumor region on CT images. The feature extraction methods proposed in Aerts et al.’s study [2] and Song et al.’s study [3] were used. For each patient, 356 three-dimensional phenotypic features and 236 two-dimensional phenotypic features were automatically extracted on the tumor regions by C++ program. More than fifty-six thousand phenotypic features were finally extracted on the 96 chemotherapy patients in this study. First, the correlation between TTP and each phenotypic feature was explored by univariate Cox regression analysis. The features identified as significant in the univariate analysis were subsequently fed into the least absolute shrinkage and selection operator (LASSO) Cox regression to build an image-based prognostic signature. The cut-off value of the signature was determined by X-tile, which was a widely recognized tool for calculating optimal cut-off values (Yale University School of Medicine, New Haven, CT, USA) [4]. Patients with different signature score would be classified into different groups according to the cut-off value. The difference of TTP in the groups classified by the signature was described by Kaplan-Meier survival curves. Log-rank test was applied to evaluate the survival difference between groups.

**Reference**

1. Yushkevich PA, Gerig G (2017) ITK-SNAP: An Intractive Medical Image Segmentation Tool to Meet the Need for Expert-Guided Segmentation of Complex Medical Images. IEEE Pulse 8:54–57
2. Aerts HJ, Velazquez ER, Leijenaar RT et al (2014) Decoding tumour phenotype by noninvasive imaging using a quantitative radiomics approach. Nat Commun 5:4006
3. Song J, Liu Z, Zhong W et al (2016) Non-small cell lung cancer: quantitative phenotypic analysis of CT images as a potential marker of prognosis. Sci Rep 6:38282
4. Camp RL, Dolled-Filhart M, Rimm DL (2004) X-tile: A new bio-informatics tool for biomarker assessment and outcome-based cut-point optimization. Clin Cancer Res 10:7252–7259

**Part 2: Image-based prognostic signature**

*Signature* = 5.654121e-11 × *Value of ClusterShade of Co-occurrence[0,1]*

-2.726245e-04 × *Value of Variance of First-order feature[1]*

-1.980131e+01 × *Value of LowGrayLevelRunEmphasis of Run-Length[3,4]*

-3.909510e-03 × *Value of GMTRvariance of GWFeature[8]*

-1.039332e-17 × *Value of GMTRvariance of GWFeature[12]*

-1.021795e-05 × *Value of GMTRvariance of GWFeature[24]*

+6.648282e-02 × *Value of GPTRentropy of GWFeature[26]*

-5.733282e-11 × *Value of GMTRvariance of GWFeature[28]*

For feature extraction, first, wavelet transform was performed on the original image and divided it into four images (LL, IH, HL, HH), which corresponding to the image 0 to image 3. The first number in the brackets of the features of *ClusterShade of Co-occurrence[0,1]* and *LowGrayLevelRunEmphasis of Run-Length[4,4]* represents these features was extracted from image 0 (LL image) and image 3 (HH image), respectively. Feature of *Variance of First-order feature* was extracted from the image 1, which represents the variance of the voxels in the region of interest of the tumor. Forty Gabor feature sets were extracted from eight directions ($\theta$= 0°, 45°, 90°, 135°, 180°, 225°, 270° and 325°) and five scales (scale = 1, 2, 3, 4, and 5 steps), which corresponding to the *GWFeature[0]~ GWFeature[39]*, The details were as follows.

**Cluster Shade of Co-occurrence:**

$$ClusterShade=\sum_{i=1}^{N(g)}\sum_{j=1}^{N(g)}\left[i+j-\chi_{x}(i)-\chi_{y}(j)\right]M(i,j)$$

Where the gray-level co-occurrence matrix is defined as$M(i,j,D,\theta)$, a matrix to describe the gray level distribution by a distance of $D$ pixels in direction $\theta$ of an image with the size of$N(g)\times N(g)$, where the $(i,j)$th element represents the number of times the combination of intensity levels occurs in two pixels in the image. The other definitions are described as follows:

$M(i,j)$ is the co-occurrence matrix by the $D$ ($D$ = 1 in this study) and $\theta$ ($\theta$ = 0°, 45°, 90°, 135° in this study),

$N(g)$ is the number of discrete intensity levels in the image,

$\chi$ is the mean of $M(i,j)$,

$\chi_{x}$ is the mean of $m_{x}$,

$\chi_{y}$ is the mean of $m_{y}$,

$\sigma_{x}$ is the standard deviation of $m_{x}$,

$\sigma_{y}$ is the standard deviation of $m_{y}$,

$p_{x}\left(i\right)=\sum_{j=1}^{N(g)}M(i,j)$ is the marginal probability of $i$,

$p_{y}\left(j\right)=\sum_{i=1}^{N(g)}M(i,j)$ is the marginal probability of $j$,

**Low-Gray-Level-Run-Emphasis of Run Length**

$$LGLRERL\text{=}\frac{\sum_{i=1}^{N(g)}\sum_{j=1}^{N(r)}\left[\frac{M(i,j|\theta)}{i^{2}}\right]}{\sum_{i=1}^{N(g)}\sum_{j=1}^{N(r)}M(i,j|\theta)}$$

Run-length is a metrics to quantify gray level runs in an image. Since the consecutive pixels that have the same gray level value in one direction could be measured, the gray level run is defined as the length in number of pixels. In a gray level run length matrix$M\left(i,j|\theta\right)$, the $(i,j)$th element describes the number of times the $j$ gray level $i$ appears consecutively in the direction specified by $\theta$. The other definitions are described as follow:

$M\left(i,j|\theta\right)$ is the $(i,j)$th point in the given run-length matrix $M$ for a direction $\theta$,

$N(g)$ is the number of discrete intensity values in the image,

$N(r)$ is the number of different run lengths,

$N(p)$ is the number of voxels in the image.

Gabor filter is a linear filter used for edge detection, which is usually used in the field of face recognition. It could select valuable image information in different directions and different scales. We used eight directions ($\theta$= 0°, 45°, 90°, 135°, 180°, 225°, 270° and 325°) and five scales (scale = 1, 2, 3, 4, and 5 steps) to extract Gabor features. Mean, variance, and entropy were used to construct the Gabor feature group. Gabor magnitude texture representation (GMTR) and Gabor phase-based texture representation (GPTR) are captured using the convolution between multi-scale and multi-directional Gabor wavelet function, and the feature we selected on the image in every direction and every scale. Here we use eight directions and five scales. $Nl$ indicates the length of histogram of gray-level of Gabor image, $P\left(i\right)$ denotes the number of gray level $i$, $N$ indicates the sum of image pixels and the $X\left(i\right)$ presents the intensity of $i$ on the Gabor image.

**Variance of Gabor**

$$Variance=\frac{1}{N-1}\sum_{i}^{N} \left(X\left(i\right)-Mean\right)^{2}$$

**Entropy of Gabor**

$$Entropy=\sum_{i=1}^{Nl} P\left(i\right)\log_{2}P\left(i\right)$$

**Part 3: The packages of R software used in this study**

R software version 3.2.3 was used in this study. The packages we used in this study are described as following.

1. LASSO Cox regression was performed using the “glmnet” package, we use the parameter of “lambda.1se” to select key features.
2. The models by multivariate Cox regression, and corresponding nomograms, and calibration plots were constructed with the “rms” package. “cph” was used to perform the Cox regression.
3. Kaplan-Meier survival analysis was performed by the “KMsurv” and “survminer” packages.
4. C-index was performed using the “Hmisc” package.
5. Decision curve analysis was performed by the “dca” package, and the net reclassification improvement (NRI) and integrated discrimination improvement (IDI) were performed by the “survIDINRI” packages, and “IDI.INF.OUT()” was used to present the results.
6. Kruskal-Wallis test was performed using the “kruskal.test()” function.

**Supplementary Table S1.** Cut-off values of blood-based biomarkers and the corresponding median of time to progression of the chemotherapy patients in this study. The default units to describe each indicator in clinical practice were used in this study.

| Blood test index | Normal value range | Number of normal/risk patients | TTP of normal/risk patients (months) |
| --- | --- | --- | --- |
| WBC | 3.5~9.5 (10^9^/L) | 52/15 | 4.1/3.3 |
| NE | 1.8~6.3 (10^9^/L) | 49/18 | 4.0/3.2 |
| LY | 1.1~3.2 (10^9^/L) | 54/13 | 3.6/3.6 |
| MONO | 0.1~0.6 (10^9^/L) | 35/32 | 3.2/3/9 |
| EO | 0.02~0.52 (10^9^/L) | 59/8 | 3.6/3.2 |
| HB | 115~150 (g/L) | 52/15 | 3.7/3.4 |
| PLT | 125~530 (10^9^/L) | 58/9 | 3.6/3.4 |
| ALT | 7~40 (U/L) | 58/9 | 4.1/1.9 |
| TBIL | 3.4~20.5 (μmol/L) | 64/3 | 3.6/4.7 |
| ALB | 40.5~55 (g/L) | 11/56 | 4.3/3.5 |
| AST | 13~35 (U/L) | 52/15 | 4.2/1.9 |
| FG | 2~4 (g/L) | 13/54 | 4.7/3.4 |
| TP | 65~85 (g/L) | 36/31 | 3.9/2.7 |
| CEA | 0~4.3 (ng/ml) | 50/17 | 3.9/3.2 |

WBC: White blood bell, NE: Neutrophil, LY: Lymphocyte, MONO: Monocytes, EO: Eosinophils, HB: Hemoglobin, PLT: platelet, ALT: Alanine aminotransferase, TBIL: Total bilirubin, ALB: Albumin, AST: Aspartate aminotransferase, FG: Fibrinogen, TP: Total protein, CEA: Carcinoembryonic antigen

**Supplementary Table S2.** Univariate Cox regression of the 24 clinical and blood-based bio-markers according to the secondary end-point of overall survival. * represents the factor was significantly associated with overall survival.

| Factors | β | Wald | HR | 95% CI | P value |
| --- | --- | --- | --- | --- | --- |
| Gender | -0.16 | 0.22 | 0.85 | 0.44 - 1.66 | 0.64 |
| Age | 0.14 | 0.29 | 1.15 | 0.68 - 1.95 | 0.59 |
| EOCG | 0.75 | 1.88 | 2.12 | 0.72 - 6.23 | 0.17 |
| Number of smoke | <0.001 | 0.77 | 1.00 | 1.00 - 1.00 | 0.38 |
| Smoke status | -0.19 | 0.47 | 0.83 | 0.48 - 1.42 | 0.50 |
| History of lung cancer | 0.12 | 0.26 | 1.13 | 0.71 - 1.80 | 0.61 |
| Family history | 0.40 | 1.43 | 1.48 | 0.78 - 2.84 | 0.23 |
| WBC | 0.59 | 5.02 | 1.81 | 1.08 - 3.04 | 0.025* |
| NE | 0.40 | 2.67 | 1.50 | 0.92 - 2.43 | 0.10 |
| LY | -0.44 | 2.04 | 0.65 | 0.35 - 1.18 | 0.15 |
| MONO | -0.38 | 2.77 | 0.68 | 0.43 - 1.07 | 0.10 |
| EO | 0.53 | 2.40 | 1.70 | 0.87 - 3.33 | 0.12 |
| HB | 0.10 | 0.15 | 1.10 | 0.67 - 1.81 | 0.70 |
| PLT | -0.02 | 0.01 | 0.98 | 0.55 - 1.75 | 0.94 |
| ALT | 0.33 | 1.31 | 1.40 | 0.79 - 2.47 | 0.25 |
| TBIL | 0.08 | 0.02 | 1.08 | 0.39 - 2.98 | 0.87 |
| ALB | -0.04 | 0.02 | 0.96 | 0.53 - 1.75 | 0.89 |
| AST | 0.69 | 7.16 | 2.00 | 1.20 - 3.32 | 0.007* |
| FG | 0.64 | 3.77 | 1.89 | 0.99 - 3.59 | 0.06 |
| TP | 0.19 | 0.67 | 1.21 | 0.77 - 1.89 | 0.41 |
| CEA | 0.37 | 2.14 | 1.45 | 0.88 - 2.40 | 0.14 |
| T stage | 0.43 | 2.89 | 1.54 | 0.94 - 2.52 | 0.09 |
| N stage | -0.15 | 0.21 | 0.867 | 0.47 - 1.60 | 0.64 |
| M stage | 0.36 | 2.43 | 1.43 | 0.91 - 2.24 | 0.12 |

WBC: White blood cell, NE: Neutrophil, LY: Lymphocyte, MONO: Monocytes, EO: Eosinophils, HB: Hemoglobin, PLT: platelet, ALT: Alanine aminotransferase, TBIL: Total bilirubin, ALB: Albumin, AST: Aspartate aminotransferase, FG: Fibrinogen, TP: Total protein, CEA: Carcinoembryonic antigen. Median of number of smoke (9600 cigarettes) was used as the cut-off value. TNM stage was divided into three variables to analysis.

**Supplementary Table S3.** The comparison of the demographics variables in three PIM subgroups.

| Factors | P value |
| --- | --- |
| Gender | 0.218 |
| Age | 0.814 |
| ECOG | 0.080 |
| Number of smoke | 0.305 |
| Smoke status | 0.374 |
| History of lung cancer | 0.694 |
| Family history | 0.064 |
| T stage | 0.493 |
| N stage | 0.425 |
| M stage | 0.710 |


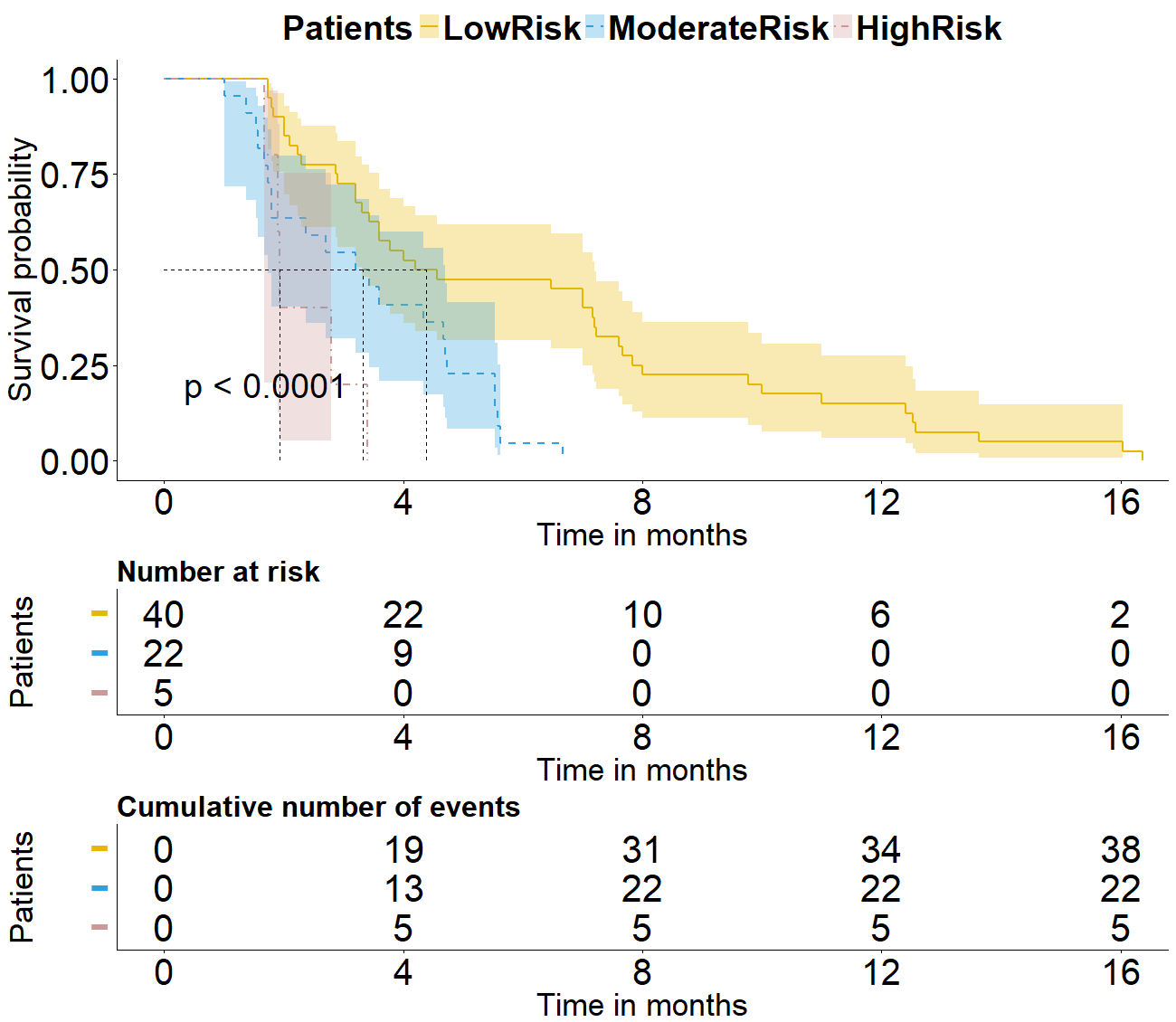


**Supplementary Fig.S1.** TTP stratification of the clinical factor-based model (AST and CEA). The result of low-risk (risk factor = 0), intermediate-risk (risk factor = 1), and high-risk (risk factor = 2) chemotherapy subgroups stratified by the model was presented. The accuracy comparison of TTP prediction between the PIM and the clinical model was statistical significant (NRI: 0.627, 95% CI: 0.310-0.762, p = 0.027 and IDI: 0.109, 95% CI: 0.015-0.203, p = 0.020).


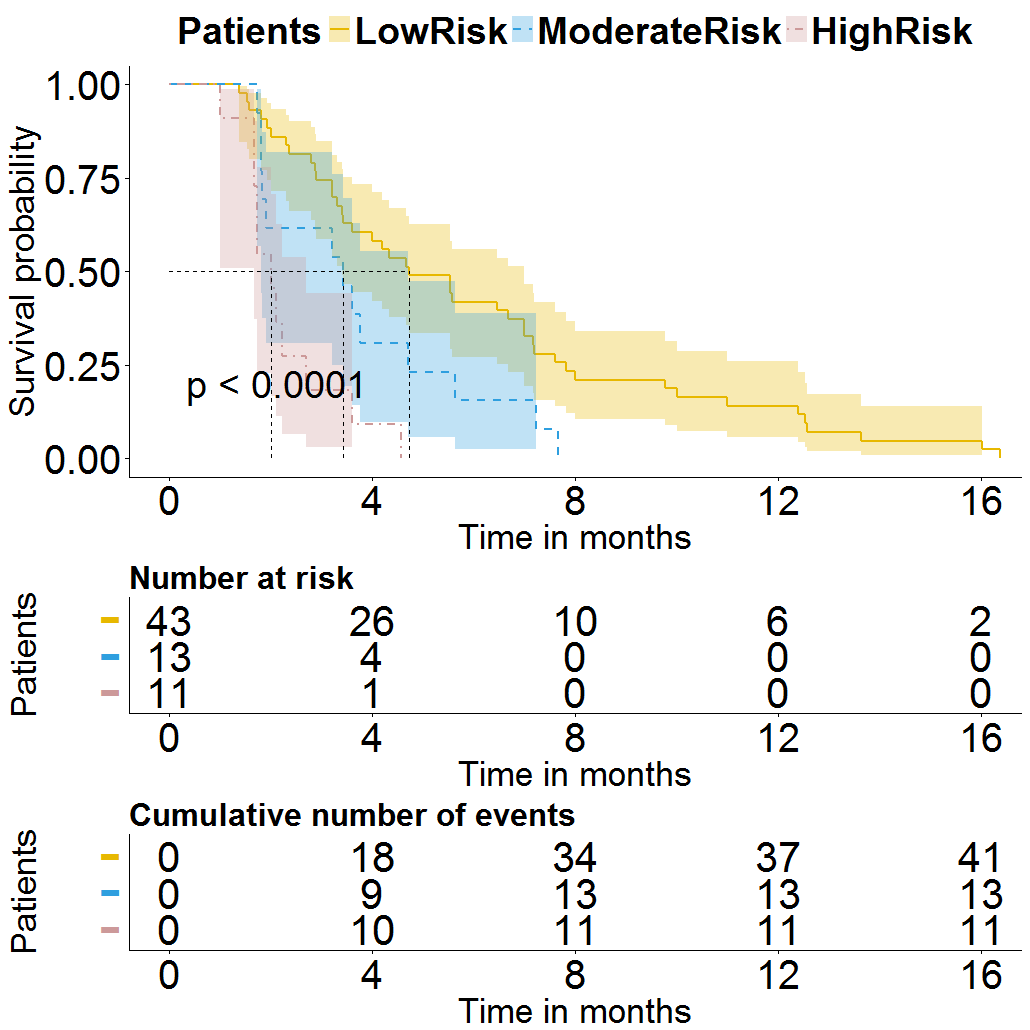


**Supplementary Fig.S2.** TTP stratification by the signature, the cut-off values were calculated by X-tile. The result of low-risk (signature score <= -1.06), intermediate-risk (-1.06 < signature score <= -0.84), and high-risk (signature score > -0.84) chemotherapy subgroups stratified by the signature were presented. The accuracy comparison of TTP prediction between the PIM and the signature was statistical significant (IDI = 0 .115, 95% CI: 0.044-0.202, p < 0.001, and NRI = 0.611, 95% CI: 0.330-0.744, p = 0.007).


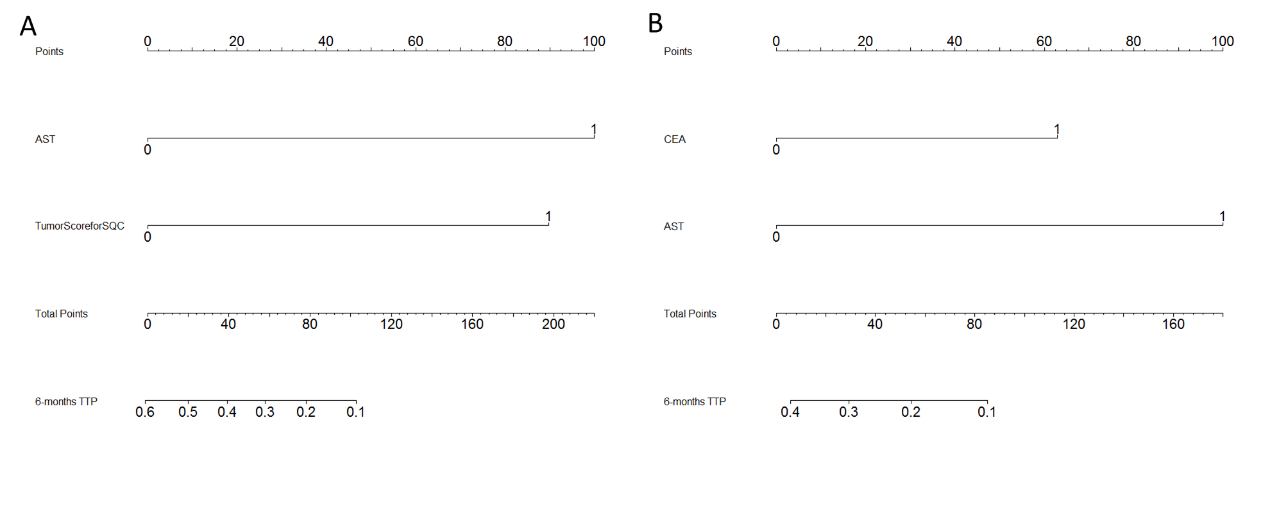


**Supplementary Fig.S3.** Nomograms of the PIM (A) and the clinical factor-based model (B). TumorScoreforSQC represents the signature score, and AST represents the Aspartate aminotransferase.


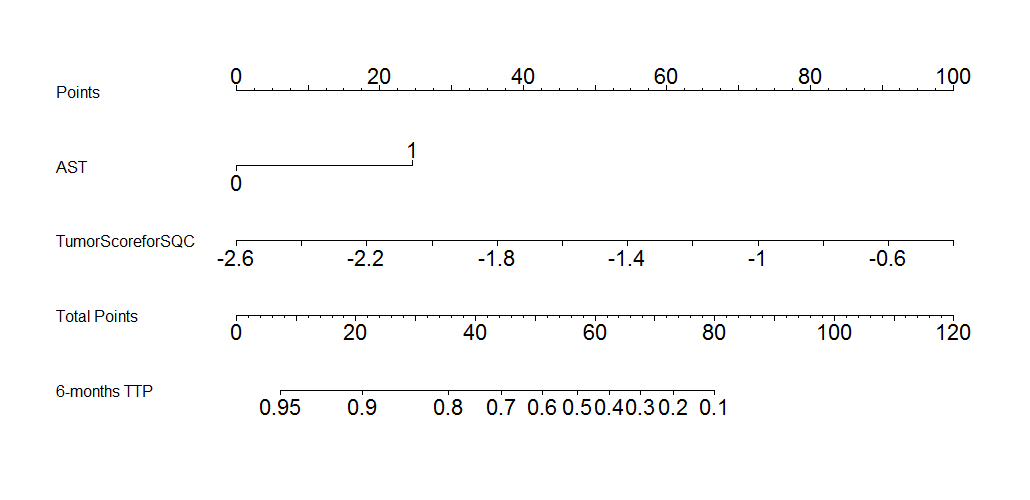


**Supplementary Fig.S4.** Nomogram of model constructed by the continuous signature and the independent significant clinical factor. TumorScoreforSQC represents the signature score, and AST represents the Aspartate aminotransferase.
